# Upregulation of cyclin D1 can act as an independent prognostic marker for longer survival time in human nasopharyngeal carcinoma

**DOI:** 10.1002/jcla.23298

**Published:** 2020-07-22

**Authors:** Yijun Liu, Qingluan Liu, Zhicheng Wang, Meilin Chen, Yi Chen, Xiayu Li, Donghai Huang, Songqing Fan, Wei Xiong, Guiyuan Li, Wenling Zhang

**Affiliations:** ^1^ Department of Medical Laboratory Science The Third Xiangya Hospital Central South University Changsha China; ^2^ The Key Laboratory of Carcinogenesis and Cancer Invasion of the Chinese Ministry of Education Cancer Research Institute Central South University Changsha China; ^3^ Xiangya Hospital Central South University Changsha China; ^4^ Department of Pathology The Second Xiangya Hospital Central South University Changsha China

**Keywords:** cyclin D1, marker, nasopharyngeal carcinoma, prognosis, survival rate

## Abstract

**Background:**

Cyclin D1 is an essential part of oncogenic transformation. We previously proved that cyclin D1 was upregulated in nasopharyngeal carcinoma (NPC) and promoted the NPC cell proliferation. But the association between cyclin D1 and the clinical outcome of NPC has not yet been determined. The study explores the possible relevance between the cyclin D1 expression and clinical parameters and its predictive value of prognosis in NPC patients.

**Methods:**

We analyzed the clinical data from 379 NPC patients and 112 non‐NPC patients in our previous study, which made further statistics. Receiver operating curve (ROC) was applied to select the optimal cutoff points. By analyzing the clinical data from 101 NPC patients using Chi‐squared test, we estimated the relationship between the cyclin D1 expression level and clinicopathological parameters. We also used Kaplan‐Meier method and log‐rank test assess and compared the disease‐free survival (DFS) rate and overall survival (OS) rate. The Cox proportional hazards model was adopted to perform the univariate and multivariate analyses.

**Result:**

Receiver operating curve analysis reported that cyclin D1 was used to differentiate between NPC patients and non‐NPC patients (*P* < .001, sensitivity: 53.6%, specificity: 85.7%, AUC = 0.752). Cyclin D1 was positively correlated with lymph node metastasis (*P* = .015). A survival analysis of the 101 NPC patients indicated that the positive expression of cyclin D1 was predictive of a good prognosis (DFS: *P* = .010, OS: *P* = .019). Multivariate analysis showed that cyclin D1 could be used independently to predict NPC patients' prognosis (DFS: *P* = .038).

**Conclusion:**

The overexpression of cyclin D1 is a good prognostic marker for NPC.

## 1 INTRODUCTION

Nasopharyngeal carcinoma (NPC) is one of the most commonly seen head and neck malignancy with high prevalence in Asia, especially in China.^1^, ^2^ Such unique ethnic and geographical distribution of NPC suggests that the causes of this disease may be linked to the genetic and environment factors.^3^, ^4^ Although great progress has been made in early diagnosis and treatment for NPC, the mortality rates still remain high as a result of local recurrences and distant metastasis.^5^, ^6^, ^7^, ^8^ Moreover, many biomarkers of NPC are undetected, and the factors related to prognosis of NPC are still unclear. Thus, the discovery of new biomarkers for NPC is helpful to the development of prognosis and treatment strategies.

The abnormal G1‐S phase in a cell cycle is one of the significant characteristics of cancer. The G1/S abnormality usually occurs in most epithelial tumors, which may lead to growth advantages and increase the occurrence of tumors.^9^, ^10^ Cyclin D1 is encoded by CCND1, which is located on chromosome 11q13. As a known regulator, cyclin D1 binds with cyclin‐dependent kinase (cdk 4/6) and inhibits the activation of retinoblastoma (Rb) protein, thus regulating the process from G1 to S phase. Accumulating researches have reported that cyclin D1 involved in cell cycle control, transcription regulation, and DNA repair.^11^, ^12^ The overexpression of Cyclin D1 may, directly and indirectly, affect the other cellular processes, thus promote the development and progression of cancers. Previously, we have proved that cyclin D1 was evidently upregulated in NPC and positively related with its progression.^13^ However, the relationship between the expression of cyclin D1 and clinical outcome of NPC is still not clear. Thus, in this research, we analyzed the clinical parameters and outcome of NPC, and provided a theoretical basis for the prognosis evaluation of NPC by analyzing the prognostic factors.

## 2 METHODS

### 2.1 Patients

Before the study we have obtained the ethical approval from Xiangya Hospital in Changsha and the Central South University Ethics Review committees/Institutional Review Boards. From January 2002 to October 2004, informed consent was obtained from all patients, that is, 112 non‐NPC cases and 379 NPC cases at Xiangya Hospital. Clinical information, including sex, age, tumor stage, lymph node metastasis, and clinical tumor‐node‐metastasis (TNM) stage, was recorded in a database.

### 2.2 Survival analysis

Valid follow‐up data of the 101 NPC patients was collected on June 30, 2019 and the correlation between the expression of cyclin D1 and clinicopathological parameters is presented in Table 1. The longest survival time was 182 months. Overall survival (OS) refers to the time from the first diagnosis to the death of any cause. Disease‐free survival (DFS) refers to the time from the diagnosis to the first treatment failure. We used Kaplan‐Meier method to estimate the OS and DFS, and the log‐rank test to compare the differences. If *P* < .05, the results analyzed in the log‐rank test were considered statistically significant. Cox regression analysis, which could be used to detect independent predictors of survival using a stepwise enter method, was carried out to examine the effect of age, gender, TNM stage, lymph node metastasis, and the expression of cyclin D1 on the clinical outcome. In a two‐tailed test, the result was considered statistically significant when *P* < .05.

**Table 1:** Clinical characteristics of patients with NPC

Parameters	Total patients (n, %)
Patient characteristics	
Median age (year)	60 (20‐74)
Age (year)	
<48	44 (43.5%)
≥48	57 (56.5%)
Gender	
Male	85 (84.2%)
Female	16 (15.8%)
Histological type	
NKC	87 (86.1%)
UC	14 (13.9%)
Clinical stage	
Ⅰ	8 (7.9%)
Ⅱ	38 (37.6%)
Ⅲ	33 (32.7%)
Ⅳ	22 (21.8%)
Tumor stage	
T1	16 (15.8%)
T2	47 (46.5%)
T3	20 (19.8%)
T4	18 (17.9%)
Lymph node metastasis	
N0	46 (45.5%)
N1	34 (33.7%)
N2	16 (15.8%)
N3	5 (5.0%)

Abbreviations: NKC, non‐keratinizing carcinoma; UC, undifferentiated carcinoma.

### 2.3 Statistical analysis

Chi‐squared test was used to examine the relationship between the expression of cyclin D1 and clinicopathological parameters. Receiver operating curve (ROC) showed the sensitivity and specificity of a test according to a scoring system for a range of cutoff point. For cyclin D1 expression, the area under the ROC curve, as a performance indicator for sensitivity and specificity, was assessed. The SPSS 20.0 statistical software (SPSS Inc) was used for calculations. If *P* < .05 in a two‐tailed test, the results would be considered statistically significant.

## 3 RESULT

### 3.1 Correlations between the expression of cyclin D1 and clinicopathological parameters in NPC patients

Table 2 showed the relationship between clinicopathological parameters of NPC and the expression of cyclin D1. Cyclin D1 expression was positively correlated with lymph node metastasis (*P* = .015). However, no association between the cyclin D1 expression and age, sex, histological type, tumor stage, and clinical stage was found.

**Table 2:** Association between CyclinD1 expression and clinicopathological parameters in NPC patients

Variables	Number	cyclinD1		*χ* ^2^	*P* Value
		Positive	Negative		
Age (year)					
<48	44	39	5	0.481	.488
≧48	57	54	3		
Gender					
Male	85	79	6	0.055	.814
Female	16	14	2		
Histological type					
NKC	87	80	7	0.014	.906
UC	14	13	1		
Clinical stage					
Ⅰ	8	8	0	2.104	.551
Ⅱ	38	34	4		
Ⅲ	33	30	3		
Ⅳ	22	21	1		
Tumor stage					
T1	16	14	2	4.641	.200
T2	47	44	3		
T3	20	17	3		
T4	18	18	0		
Lymph node metastasis					
N0	46	46	0	10.464	.015
N1	34	29	5		
N2	16	14	2		
N3	5	4	1		

Abbreviations: NKC, non‐keratinizing carcinoma; UC, undifferentiated carcinoma.

### 3.2 Cyclin D1 could be used to distinguish NPC and non‐NPC patients

**Figure 1:**
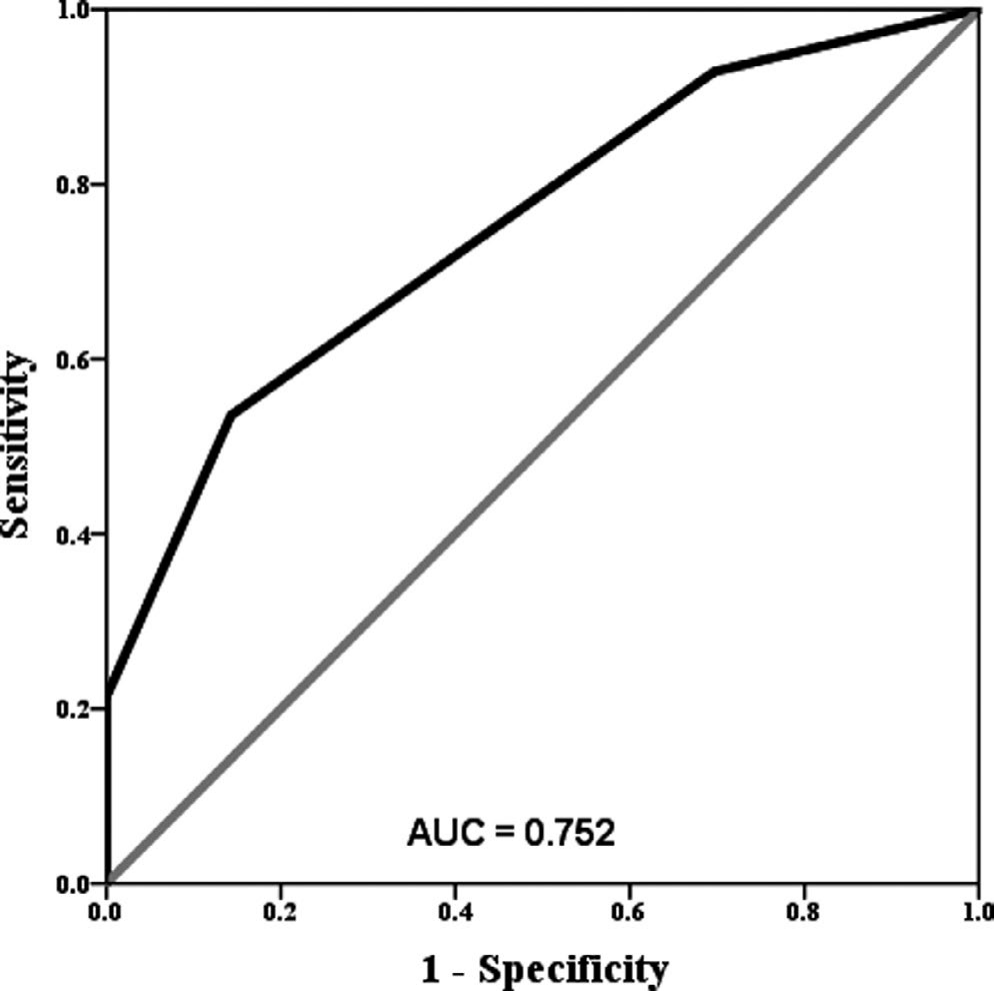
Cyclin D1 expression patterns can distinguish the non‐cancerous group from the NPC group. RPC curve analysis of cyclin D1 expression. The AUC of cyclin D1 for predicting non‐cancerous patients was 0.752, with a sensitivity and specificity of 53.6% and 85.7%, respectively

The value of cyclin D1 expression in distinguishing between NPC and non‐NPC patients was assessed. An optimal cutoff value was set to test some important date points, and then the area under the curve (AUC), sensitivity, and specificity were calculated. The ROC curve for the scoring systems for all NPC and non‐NPC patients was shown in Figure 1. The AUC for cyclin D1 expression in the prediction of NPC patients was 0.752 (95% confidence interval = 0.705‐0.798, *P* < .001), with a sensitivity of 53.6%, and specificity of 85.7%.

### 3.3 Cyclin D1 expression could be used to predict the clinical outcome of NPC

**Figure 2:**
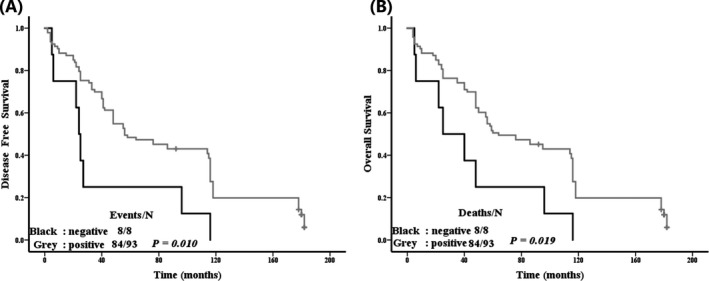
Kaplan‐Meier survival curves in NPC patients with varying cyclin D1 expression. Cyclin D1 expression levels differentiate DFS and OS in the two patient groups: positive expression (gray) and negative expression (black). DFS: *P* = .010; OS: *P* = .001 (log‐rank test)

We have obtained valid follow‐up data from 101 NPC patients. Then Kaplan‐Meier survival analysis was used to examine the association between the cyclin D1 expression and cancer‐related deaths. The results suggested that the expression of cyclin D1 significantly affects the DFS and OS of NPC patients (Figure 2). Moreover, univariate survival analysis found that the patient histological type, clinical stage, tumor stage, and lymph node metastasis are also related to the prognosis of the patients (Table 3, all *P* < .05). To evaluate whether the predictive value of cyclin D1 expression for the prognosis of NPC is related with other clinicopathological features of NPC patients, we carried out multivariate analysis for age, histological type, lymph node metastasis, tumor stage, clinical stage, cyclin D1 negative expression, and positive expression. According to the results, lymph node metastasis (DFS: *P* = .008; OS: *P* = .02), tumor stage (DFS: *P* = .000; OS: *P* = .000), and cyclin D1 (DFS: *P* = .038) were powerful independent predictors of NPC patient survival (Table 4).

**Table 3:** Univariate survival analysis of DFS and OS in 101 NPC patients

Variable	DFS			OS		
	Exp (B)	95% CI	*P* value	Exp (B)	95% CI	*P* value
Age (<48 y/≧48 y)	0.974	0.788‐1.203	.806	0.981	0.794‐1.212	.860
Gender	0.798	0.464‐1.371	.413	0.817	0.475‐1.405	.465
Histological type	1.462	1.061‐2.015	.020	1.509	1.096‐2.077	.012
Clinical stage (Ⅰ+Ⅱ/Ⅲ+Ⅳ)	1.020	1.001‐1.039	.042	1.021	1.002‐1.041	.031
Tumor stage (T1 + T2+T3/T4)	0.542	0.408‐0.722	.000	0.528	0.396‐0.703	.000
Lymph node metastasis (N0 + N1+N2/N3)	0.737	0.597‐0.910	.004	0.742	0.601‐0.915	.005
Cyclin D1 (Negative/Positive)	0.993	0.987‐0.999	.016	0.993	0.987‐0.999	.026

**Table 4:** Significant prognostic factors affecting DFS and OS as determined by multivariate analysis (Cox regression)

Variable	DFS			OS		
	Exp (B)	95% CI	*P* value	Exp (B)	95% CI	*P* value
Age (<48 y/≧48 y)	0.694	0.437‐1.1	.12	0.719	0.456‐1.136	.157
Histological type	1.567	0.75‐3.274	.232	1.686	0.081‐3.513	.163
Clinical stage (Ⅰ+Ⅱ/Ⅲ+Ⅳ)	1.712	0.913‐3.208	.093	1.594	0.847‐2.999	.148
Tumor stage (T1 + T2+T3/T4)	0.189	0.094‐0.378	.000	0.192	0.096‐0.385	.000
Lymph node metastasis (N0 + N1+N2/N3)	0.448	0.248‐0.81	.008	0.495	0.274‐0.897	.02
cyclinD1 (Negative/Positive)	2.446	1.052‐5.688	.038	2.257	0.974‐5.229	.058

## 4 DISCUSSION

Nasopharyngeal carcinoma (NPC) is an epithelial malignancy with special characteristics with regard to ethnicity and geography. The development of NPC is resulted from complex interactions between environmental and genetic factors, and its progression has complex caused.^14^ The major issues of poor survival in NPC are distant metastasis and local recurrence. In the process of studying cyclin D1, the earliest known function of the gene is to form a complex with CDK4 or CDK6, which regulates the cell cycle G1/S transformation to promote cell proliferation.^15^, ^16^ Thus, cyclin D1 overexpression would loss control of normal regulatory constraints and affect cellular processes, which may lead to tumorigenesis. Furthermore, cyclin D1 acts as an oncogene, which profoundly affects the tumor microenvironment promoting tumor metastasis and increasing angiogenesis.^17^, ^18^, ^19^ Moreover, cyclin D1 overexpression is also involved in multiple signaling pathways, including JUK, and NF‐kB pathways.^13^ Accumulating studies revealed that cyclin D1 was upregulated in various cancers, including breast cancer, melanoma, colorectal cancer, lung cancer, oral squamous cell carcinoma, as well as NPC^20^, ^21^, ^22^; Thus, it might be indicative of the clinical prognosis in NPC.

Recently, Hur et al found a significant relevance between cyclin D1 expression and aggressive tumor histology, lymph node metastasis, and higher AJCC stages in squamous cell carcinoma.^23^ A similar study on mantle cell lymphoma suggested that patients with cyclin D1 overexpression had worse prognosis.^24^ In addition, cyclin D1 overexpression has been proved to be related to poor prognosis in patients with cancers like lung cancer and oropharyngeal cancer.^25^, ^26^ In contrast, studies found that increased levels of cyclin D1 expression was associated with positive prognosis in breast cancer.^27^, ^28^ These researches indicated that cyclin D1 may play different roles in the prognosis of cancer. But the mechanism of cyclin D1 survival prognosis is unknown.

Our previous research had demonstrated that cyclin D1 was overexpressed in NPC. However, the association between the expression of cyclin D1 and clinicopathological parameters was not clearly. In this study, we analyzed the association between cyclin D1 and clinicopathological parameters, and extended the follow‐up period of NPC patients. No significant correlation between cyclin D1 expression and patients’ age gender, histological type, tumor stage, and clinical stage was observed in this study, and similar results were reported by many previous studies.^29^, ^30^ However, a significant positive correlation between cyclin D1 overexpression and lymph node metastasis was found in our study, which was consistent with other studies.^31^ ROC analysis was performed to verify the ability of cyclin D1 expression in distinguishing between non‐NPC patients and NPC patients, which has not been reported in other studies. The Kaplan‐Meier survival analysis showed that cyclin D1 could be used as a prognostic biomarker for NPC. Through univariate and multivariate analysis, we further revealed that tumor stage, lymph node metastasis, and cyclin D1 positive expression were highly correlated with the prognosis of NPC patients. These data proved that cyclin D1 was an independent prognostic biomarker for NPC.

Mylona et al suggested that the underlying mechanisms of the association between cyclin D1 and good prognosis could be interpreted by the interaction between cyclin D1, histone acetylases, and Rb, since Rb acetylation inhibits the cell cycle and growth.^32^ Moreover, Bustany et al revealed that cyclin D1 could activate the unfolded protein responses and induce endoplasmic reticulum stress‐mediated apoptosis.^33^ Because the activation of oncogene increases the bioenergy process, cancer cells would experience intrinsic stress. Thus, cancer cells need to adapt to the intrinsic stress and to keep surviving and growing. The overexpression of cyclin D1, as a frequent phenomenon in many tumors, could enhance protein synthesis and recombine of metabolic pathways to meet the demands of rapid cell growth and proliferation. However, in the development of tumors, the protein synthesis needs to be maintained in a balanced level, so that the cancer cells can proliferate normally without undergoing reticulum stress. Otherwise, it can cause protein toxicity and block tumorigenesis. Therefore, we could further explain that cyclin D1 overexpression may promote malignant progression and induce robust anabolic and proliferative programs, and then cause intrinsic stress, which would lead to proteotoxicity and block tumorigenesis. In addition, genomic DNA damage may affect the susceptibility to NPC and be related with NPC progression,^34^, ^35^, ^36^ so cyclin D1 as the positive prognostic marker in NPC could be interpreted as related to the amplification and deletion of other genes.

In conclusion, this study proves that cyclin D1 expression had the strongest correlation with lymph node metastasis and could help to distinguish NPC and non‐NPC patients. Furthermore, the overexpression of cyclin D1 in NPC is closely association with good prognosis in NPC patients. However, the specific mechanism of how cyclin D1 expression relates to prognosis in NPC patients has not been elucidated. Thus, we need to explore this mechanism in further studies.

## CONFLICT OF INTEREST

We wish to draw the attention of the editor to the following facts which may be considered as potential conflicts of interest and to significant financial contributions to this work. We confirm that the manuscript has been read and approved by all named authors and that there are no other persons who satisfied the criteria for authorship but are not listed. We further confirm that the order of authors listed in the manuscript has been approved by all of us. We confirm that we have given due consideration to the protection of intellectual property associated with this work and that there are no impediments to publication, including the timing of publication, with respect to intellectual property. In so doing we confirm that we have followed the regulations of our institutions concerning intellectual property. We understand that corresponding author is the sole contact for the editorial process (including editorial manager and direct communications with the office). She is responsible for communicating with the other authors about progress, submission of revision and final approval of proofs. We confirm that we have provided a current, correct email address which is accessible by the corresponding author.

## AUTHOR CONTRIBUTIONS

This research was conceived and designed by Wenling Zhang and Yijun Liu; materials was collected by Qingluan Liu and Zhicheng Wang. The statistical analysis and the interpretation of the results were performed by Yijun Liu. The study was reviewed and edited by Xiayu Li, Donghai Huang, Songqing Fan, Wei Xiong, Guiyuan Li, Wenling Zhang, and Yijun Liu, and the study was polished by Meilin Chen and Yi Chen. reviewed and edited the study. The final study was approved by all the authors.
